# Easy to criticize when you have not done the work: when letters to the editor become a substitute for original research

**DOI:** 10.1080/07853890.2026.2724569

**Published:** 2026-09-06

**Authors:** Filippo Migliorini, Nicola Maffulli

**Affiliations:** a Department of Trauma and Reconstructive Surgery, University Hospital of Halle, Martin-Luther University Halle-Wittenberg, Halle, Saale, Germany; b Department of Orthopaedic and Trauma Surgery, Academic Hospital of Bolzano (SABES-ASDAA), Bolzano, Italy; c Department of Life Sciences, Health, and Health Professions, Link Campus University, Rome, Italy; d Department of Orthopaedic and Trauma Surgery, Faculty of Medicine and Psychology, University La Sapienza, Roma, Italy; e School of Pharmacy and Bioengineering, Keele University Faculty of Medicine, Stoke-on-Trent, UK; f Centre for Sports and Exercise Medicine, Barts and the London School of Medicine and Dentistry, Mile End Hospital, Queen Mary University of London, London, UK

Academic medicine advances through dialogue. Every scientific article enters the literature as an implicit invitation to respond, and for decades, letters to the Editor have provided a protected space for clarification, correction, and interpretation. In orthopaedics, where surgical decision-making depends on nuance and context, this tradition has been particularly valuable, especially within sports medicine, where return-to-play decisions, performance outcomes, and time-sensitive management of athletes depend on highly specialised interpretation of evidence. Well-constructed letters can clarify methodological issues, identify limitations, and stimulate further research. This Editorial focuses on a specific concern arising from this debate: the use of Letters to the Editor as a recurrent form of academic output and its implications for scientific contribution, academic recognition, and editorial responsibility.

In recent years, however, the nature of this exchange has shifted. Journals have increasingly received extremely critical letters from individuals with no established publication record, some of whom have little or no demonstrable record of original research across major bibliographic databases. Their presence in the literature consists almost exclusively of often non-constructive or predominantly adversarial letters on the work of others. What was once the culmination of sustained scientific effort can now be achieved through brief opinion pieces that question the methodology of experienced research groups without contributing original evidence. When repeated over time, such correspondence may become a substantial component of an individual’s publication record, allowing academic visibility to be accumulated primarily through criticism rather than through the production of original research.

This trend also exposes a problem of reciprocity and academic recognition. Producing original research requires conceptual design, ethical oversight, data collection, and analytical accountability, all under the scrutiny of peer review. Writing a critical letter, by contrast, carries little comparable burden. Yet, in some academic evaluation systems, these outputs may receive similar formal recognition, with letters being counted alongside clinical studies for promotion. This creates an imbalance between intellectual risk and academic reward. The concern is not that Letters to the Editor have no academic value, but that their repeated use should not provide a shortcut to academic recognition comparable to that associated with the generation of original evidence.

The incentives are clear. Modern academic assessment prioritises numerical productivity and visibility. Letters to the Editor provide a relatively accessible route to indexed academic output and may therefore be attractive within publication-driven evaluation systems. For early-career researchers, this may represent a transitional strategy. However, when correspondence becomes the predominant form of an individual’s academic output, it risks becoming a substitute for scientific engagement rather than a complement to it. This accessibility is not inherently problematic; the concern arises when such contributions are evaluated as equivalent to original research despite their fundamentally different evidential and methodological scope.

The implications for orthopaedic and trauma surgery are not trivial. Methodological expertise is acquired through practice and experience, particularly in sports medicine, where interpretation of outcomes such as return to sport, performance recovery, and reinjury risk requires direct clinical exposure to athletic populations. Statistical interpretation, bias assessment, and contextual understanding cannot be improvised. Relevant expertise can strengthen the interpretation of complex clinical or methodological issues, but expertise should not be regarded as a prerequisite for legitimate scientific criticism. A valid criticism may arise from clinicians, researchers, statisticians, methodologists, or other readers whose contribution is supported by sound reasoning and evidence.

This imbalance also has implications for editorial processes. Original studies undergo rigorous methodological evaluation, whereas the assessment of Letters to the Editor may place greater emphasis on relevance, tone, and format than on the methodological basis of the claims being made. Yet, once indexed, these letters influence perceptions of techniques, technologies, and researchers alike and, in sports medicine, may indirectly shape clinical decisions that affect athletes’ recovery timelines, return-to-play strategies, and long-term joint health. Editorial offices, therefore, bear responsibility not only for facilitating debate but also for ensuring its credibility. Editorial offices should therefore ensure that critical correspondence is assessed for factual accuracy, methodological soundness, relevance, and conflicts of interest, while preserving its role as a forum for scientific debate. These measures should involve journal editors and publishers, as well as academic institutions responsible for research assessment and promotion.

If letters are to count as academic contributions, they should reflect an understanding of what such contributions entail. Otherwise, the literature risks drifting toward a paradox in which individuals who have never produced original evidence may be rewarded for repeatedly judging the evidence produced by others. Orthopaedics, and particularly sports medicine, is a discipline grounded in measurable actions, performance outcomes, and decisions that directly affect the careers and health of athletes. Debate should sharpen practice, not replace it. The opportunity to contribute to scientific debate should remain open, but academic recognition should be earned through substantive contributions to knowledge generation rather than through criticism alone.

